# Genome Sequences of Rhinovirus C Isolates from Wisconsin Pediatric Respiratory Studies

**DOI:** 10.1128/genomeA.00203-14

**Published:** 2014-03-27

**Authors:** Stephen B. Liggett, Yury A. Bochkov, Tressa Pappas, Robert F. Lemanske, James E. Gern, Naomi Sengamalay, Xuechu Zhao, Qi Su, Claire M. Fraser, Ann C. Palmenberg

**Affiliations:** a Department of Medicine, University of South Florida, Morsani College of Medicine, Tampa, Florida, USA; b Department of Pediatrics, University of Wisconsin School of Medicine and Public Health, Madison, Wisconsin, USA; c Institute for Genome Sciences, University of Maryland School of Medicine, Baltimore, Maryland, USA; d Institute for Molecular Virology, University of Wisconsin–Madison, Madison, Wisconsin, USA

## Abstract

Human rhinovirus (RV) isolates from the RV-C species are recently discovered infectious agents that are closely linked to asthma and wheezing etiologies in infants. Clinical study samples collected at the University of Wisconsin–Madison describe 41 nearly complete genome sequences representing 21 RV-C genotypes.

## GENOME ANNOUNCEMENT

Human rhinovirus (RV) isolates comprise the RV-A, RV-B, and RV-C species of the *Enterovirus* genus in the *Picornaviridae* family. A classic panel of 99 RV-A and RV-B species are the canonical agents of the common cold. A full set of RNA genome sequences for these historic types was completed in 2009 (1). Although serotyping played an early role in RV taxonomy, current classification is based on sequence conservation (2). Strains are assigned to common species if they share >70% amino acid identity in the P1, 2C, and 3CD regions. Isolates are further subdivided into numeric genotypes that respect the historic naming system, but now rely almost entirely on sequence comparisons of the VP1 or VP4/VP2 coding sequences. The preferred RV nomenclature designates the species letter (A, B, or C) and type number (e.g., A16). Strain designations are unique to each GenBank accession number. Assignment of a new strain to a known genotype requires >86 to 87% aligned nucleic acid identity in either or both of the key capsid-coding regions (2).

The RV-C species were first discovered in 2006 as part of broad spectrum clinical surveillance studies (3–5). While clearly rhinoviruses, they are not readily propagated in typical cell culture systems (6) so much of their biology is inferred from sequence comparisons. Currently, 51 genotypes (as binned by VP1 nucleotide identity) have been described (2). These isolates are important because they are associated with up to half of RV infections in young children (6). Within the context of virus surveillance, the University of Wisconsin hospitals and clinics in Madison, WI, are participating in several studies with the goal of determining how RV sequence variation is linked to cold symptoms and asthma exacerbations. The Childhood Origins of Asthma (COAST), Mechanisms and Environmental Determinants of Rhinovirus Illness Severity (RhinoGen), and T Regulatory Cells and Childhood Asthma (T-Reg) protocols collect and screen infant nasal secretions using multiplex PCR assays (7), rhinovirus PCR (8), or both. Between 1999 and 2010, hundreds of solitary RV infections were identified. Partial sequencing assigned these isolates to relevant species, but for some, particularly the RV-C species, the data suggested several potential new genotypes, or provided confirmation for similar reclassification proposals (9).

Multiple COAST and RhinoGen isolates were then reexamined using massively parallel sequencing techniques applied directly to clinical samples (10). The single-pass methodology gave, on average, 93% genome coverage to a depth of 8 to 10 reads for 179 study-specific isolates. For the RV-C species, the technique resolved nearly full genomes for 41 isolates, representing 21 different genotypes (9). Relative to prototype RV-C genomes, which average ~7,097 bases (b) (1), most of these assemblies were missing the difficult-to-sequence 5′ and/or 3′ termini (average, Δ 465 b) and occasionally, short internal fragments (<100 b) for which the contigs were not be explicitly linked. Nevertheless, every new sequence (average, 6,592 b; median, 6,632 b) was unambiguously aligned with an index compilation of RV-C prototype sequences (2). For C17, C22, C26, C28, C32, C36, C38, C41, C42, C43, C45, and C49, the new data include the first non-capsid descriptions of these genotypes.

### Nucleotide sequence accession numbers.

Each contiguous data set has been deposited at DDBJ/EMBL/GenBank using the accession numbers listed below. Each unit described here is the first genome version of the sequence of that isolate: CO2, JN815248, JN837695, JN990703, JQ245968, and JX025557; C03, JN798567, and JN990700; C04, JF781509; C06, JN815245, and JN990702; C07, JN798559, JN798570, JN837689, JQ994495, and JX025556; C08, JQ245964, and JQ245973; C15, JN837688; C17, JN815240, JN815244, and JQ837720; C22, JN621242; C25, JN837685; C26, JX193796; C28, JN798569; C32, JN798581, and JQ994498; C36, JN541267; C38, JN837691; C40, JF781505, JN815251, and JQ245963; C41, JN798565; C42, JQ994500; C43, JN815249, JN837687, and JX074056; C45, JN837686; C49, JF907574, JN798566, and JN798568.
